# The Mastoid Operation

**Published:** 1894-04

**Authors:** B. F. Church

**Affiliations:** First Assistant Physician North Texas Hospital for the Insane, Terrell


For Texas Medical Journal.

### THE MASTOID OPERATION.

B. F. CHURCH, M. D.,
First Assistant Physician North Texas Hospital for the Insane, Terrell.

THE MASTOID operation is understood to be the surgical procedure of opening the mastoid antrum through the mastoid cells.

While the indications for performing the operation are now pretty well established, the history of the procedure, from the time nature and accident first peformed it, to the present time, is notable for its tardiness in reaching a recognized position in surgery.

The first recorded operative opening of the mastoid cells (the antrum not being reached) was in 1649, and was performed for the relief of deafness and tinnitus caused by obstruction of the

Eustachian tube, with the idea that ventilation of the tympanum was thereby established.

One hundred years later, a Prussian military surgeon, while sounding an abscess behind the ear, accidently pushed his sound through the necrotic cortex, and in syringing was greatly astonished and terrified (not being familiar with the anatomy of the parts) at seeing the fluid pass out at the patient's nose. The operation was successful, however, the patient being relieved of a very painful affection. The results of this case spread rapidly and the operation of simply cutting into the mastoid cells was performed with variable results in a great number of ear diseases.

Among those who lost their lives from the operation was the physician to the Danish court. After that casualty public opinion quickly turned against the operation, and it was entirely abandoned.

Even up to the middle of the present century the best medical authorities persisted in their condemnation of it.

Turnbull, in 1861, reported the first successful case in this country, and in that the bone was so diseased that a probe was pushed through the thinned cortex, after the soft parts had been incised.

There exists at the present time a wide diversity of opinion among surgeons as to what symptoms constitute a positive demand for the mastoid operation. Undue conservatism, both as to the time for surgical interference and the extent of the operation, seems to prevail.

The approved method of operating is with mallet and chisels, or gouges, and sharp spoons. The incision of the soft parts should be made, beginning at one-half an inch above the upper insertion of the auricle, curve within one quarter of an inch of its posterior insertion and extend down to the tip of the mastoid process. The periosteum should be evenly divided and turned back so as to expose the spina and linea temporalis, they being the most reliable land-marks for the perforation. If no anomaly exists in the depth of the middle cerebral fossa or the position of the lateral sinus, we are safe in sinking the canal as follows:

With the largest gouge an oval area of bone, by gentle taps with the mallet, is marked out eight to ten m.m in width and about twelve m.m in length, with its upper border corresponding to the linea temporalis and the middle of the opening just posterior to the spina supra meatum. The opening should be started as close to the spina as possible without endangering it during the subsequent chiseling. Sink the canal slightly forward, nearly parallel to the meatus, using successively smaller sized gouges as its depth increases. Precede each step, after reaching the cells, by a thorough examinalion with a probe. The opening made with the gouges or chisels should be cone-shaped, so adjusted that the apex will reach the antrum. When that cavity is presumably reached, with the smallest gouge, introduce a bent probe to verify it, then with sharp spoon ream off all sharp spiculæ of bone and enlarge the opening into the antrum.

This operation, so briefly described, is much safer than the older method of trephining. It is the typical operation, or that of Schwartze, performed for the relief of middle ear suppurations, or acute cases, where the process is limited to the middle ear and antrum. Those in which carious necrosis of the cortex exists, are modified according to the conditions found, but in all cases it is best not to stop short of a free opening into the antrum, which affords a means for thorough cleansing and drainage,—a surgical requirement of the greatest import.

